# Prohibitins: emerging host targets of bacteria and viruses at the plasma membrane, mitochondria, and cytoplasm

**DOI:** 10.15698/mic2026.06.878

**Published:** 2026-06-03

**Authors:** Grecia O. Rivera-Palomino, Arianne L. Theiss

**Affiliations:** 1 Department of Immunology and Microbiology, University of Colorado – Anschutz Medical Campus, Aurora, CO, USA; 2 Division of Gastroenterology and Hepatology, University of Colorado – Anschutz Medical Campus, Aurora, CO, USA; 3 Rocky Mountain Regional Veterans Affairs Medical Center, Aurora, CO, USA

**Keywords:** infection, pathogen, host microbe interaction, mitochondrial dysfunction, infectious disease

## Abstract

Ubiquitously expressed Prohibitin-1 (PHB1) and Prohibitin-2 (PHB2) serve pleiotropic functions in cellular processes, including signal transduction, mitochondrial metabolism and dynamics, and lipid raft formation. Located in phospholipid-rich subcellular sites such as the plasma membrane and mitochondrial inner membrane, PHB1 and PHB2 are emerging as important host targets for bacteria and viruses, influencing infection and host responses by these microbes. Here, we present the current understanding of PHB1 and PHB2 in bacterial and viral infection based on the cellular localization of PHBs at the plasma membrane, mitochondria, and cytoplasm. We also discuss the potential of targeting PHBs as therapeutics for bacterial and viral infections.

## INTRODUCTION

**Figure 1:**
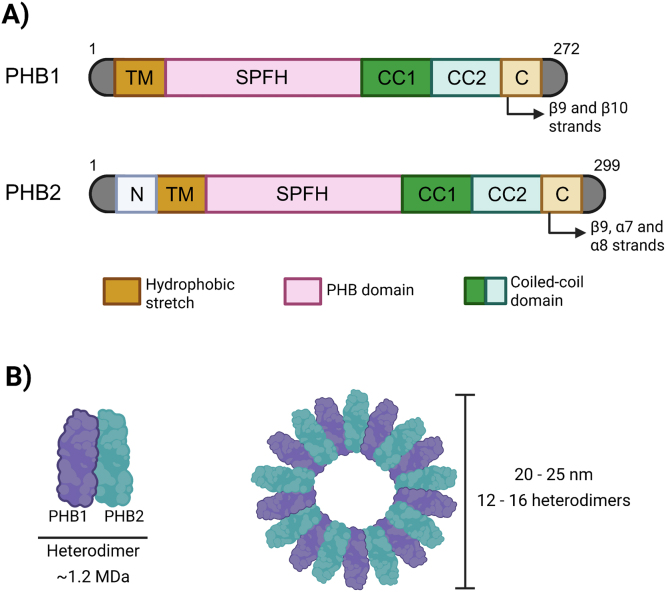
PHB protein domains and heterodimer cellular organization. **(A)** PHB1 and PHB2 share more than 50% identical amino acid residues and are highly homologous to each other. PHBs contain hydrophobic stretches that anchor the complex to the inner mitochondrial membrane at the N-terminus. Although both PHB1 and PHB2 contain a transmembrane domain (TM) at the N-terminus, PHB2 possesses a non-cleavable mitochondrial targeting sequence, which has positively charged amino acids. Conversely, PHB1 lacks this signal sequence. The PHB domain is characteristic of the SPFH-family of membrane proteins. **(B)** Some shared properties across SPFH members are self-oligomerization into large membrane complexes and scaffold roles to organize functional membrane microdomains. PHBs have 2 predicted coiled-coil domains at the carboxyl terminal end, which contribute to PHB complex assembly. The coiled-coil structures possess a leucine-isoleucine-rich nuclear export sequence (NES). The NES facilitates the export of PHB from the nucleus to the cytoplasm or mitochondria, allowing active shuttling between different organelles. Created in BioRender (Rivera, G. (2026) https://BioRender.com/vzs06xr).

Prohibitins are evolutionarily conserved and ubiquitous proteins that are localized in the eukaryotic nucleus, plasma membrane and mitochondria 1. Genetic deletion of *Phb1* or *Phb2* in mice results in embryonic lethality, suggesting these genes are essential for development 2–4. The PHB complex is formed by two highly homologous subunits, PHB1 (formerly known as BAP32), and its homolog PHB2 (formerly known as prohibitone, BAP37, or REA). PHB1 (molecular weight of 32 kDa) and PHB2 (molecular weight of 34 kDa) share 50% amino acid sequence identity and 60% similarity (Figure 1**A**) 1, 5, 6. PHB1 and PHB2 belong to the ancient and universally conserved stomatin/prohibitin/flotillin/HflK/C (SPFH) family of proteins based on the presence of the prohibitin domain and related to membrane protection and membrane fluidity regulation 6, 7.

Initially, it was proposed that the PHB complex has a role in cell cycle progression 2. Over the past ∼ 30 years, PHBs have also been implicated in cellular signaling, apoptosis, transcriptional regulation, mitochondrial biogenesis and regulation of sister chromatid cohesion 8–10. This diverse range of functions exerted by PHB1 and PHB2 is thought to depend on their subcellular localizations and post-translational modifications. In fact, various post-translational modifications, such as ubiquitination, acetylation, and phosphorylation, can be associated with specific localizations of PHBs within the cell 11, 12.

Of note, both PHB1 and PHB2 can localize in the nucleus mediated by a C-terminal nuclear import sequence in PHB2 and a C-terminal nuclear export signal in PHB1 12. The C-terminal domain in PHB1 contains β 9 and β 10 strands, whereas PHB2 has β 9, α 7 and α 8 strands 13. This nuclear localization of PHBs has been shown to regulate gene transcription via interaction with transcription factors, including p53, Rb, E2F, AIF, c-myc, or c-fos, which has been most widely characterized in cancerous cell lines 14. Here, we will discuss the main functions of PHB1 and PHB2 in the mitochondria and plasma membrane, since these locations within the host cells have been implicated in bacterial and/or viral interactions.

## SUBCELLULAR LOCALIZATION AND FUNCTIONALITY OF PHBS

### Plasma membrane

PHBs act as transmembrane adaptors to activate downstream signal transduction at the cell plasma membrane 14. One signaling cascade activated is the Raf-MEK-ERK pathway, which plays an important role in epithelial cell adhesion and migration 15. In cancer cells, PHBs are required for C-Raf association and subsequent activation of the Raf-MEK-ERK signaling pathway 15. In addition, PHBs localization at the plasma membrane in platelets, B cells and adipose tissue contributes to cell-cell communication and may function as a receptor platform for small ligands 16.

Additional functions of cell surface-localized PHBs include serving as specific receptors in white adipose vasculature and acting as lipid scaffolds that preserve the integrity of the plasma membrane 15, 17. In both humans and mice, PHBs are abundantly enriched in endothelial cells of white adipose tissue, where adipose-targeting peptides selectively bind membrane-associated PHB1 and promote its internalization 14, 18. In B-lymphocytes, PHBs function as scaffold proteins through interaction with CD86, leading to activation of NF- κ B p50/p65 signaling 19. In macrophages, PHBs regulate their inflammatory signaling by modulating fatty acid composition, thereby influencing membrane packing, lipid raft formation, and maintaining the overall plasma membrane organization 20.

Due to their localization at the plasma membrane, PHBs have been implicated in multiple infectious diseases, where they function as host factors facilitating host-pathogen interactions 16 and will be discussed in Section “Plasma Membrane PHBs and Viral and Bacterial Infections”. PHBs can act as receptors or co-receptors to promote viral entry and infectivity. Furthermore, PHBs in the plasma membrane have been associated with drug-resistant cancer cells. They act as receptors to certain molecules and modulate or participate in cell-cell communication 14, 16. These diverse functions highlight the importance of membrane-localized PHBs as dynamic regulators of cell signaling and disease progression.

### Mitochondria

PHB1 and PHB2 are located in the inner mitochondrial membrane (IMM), where they form a heterodimeric molecular complex of ∼ 1.2 MDa composed of 12–16 PHBs heterodimers, associated in a ring-like structure with a diameter of 20–25 nm 21, 22 (Figure 1**B**). Immuno-depletion and co-immunoprecipitation assays confirmed a physical interaction between PHB1 and PHB2, with both present as a complex and not as free proteins or homodimers 23, 24. A recent study from Lange *et al.* utilized cryo-electron tomography, sub-tomogram averaging and molecular modelling to propose a bell-shaped structure of 11 alternating human PHB1 and PHB2 molecules 25. This heterodimeric complex promotes interdependent functions of PHB1 and PHB2 in various organisms such as mice, *Saccharomyces cerevisiae*, *Caenorhabditis elegans* and human cell lines 3, 8.

Both PHBs are targeted to the IMM by an N-terminal transmembrane domain, considered a conserved domain common to other scaffold proteins, and that is not cleaved upon mitochondrial import 15. Tim8-Tim13 complex associates with PHB1 in the mitochondrial intermembranous space. Then, the insertion of N-terminal hydrophobic sequences of PHB1 into the IMM is mediated by Tim23 translocase, accompanied by the assembly of PHB2 subunits into 120 kDa complexes, before forming the large ring-like complexes 21. It is important to mention that only PHB2 has the N-terminus containing a predicted mitochondrial targeting sequence 22, 26. The C-terminal coiled-coil domains in PHB1 and PHB2 both expose large domains to the intermembranous space and facilitate subsequent oligomerization into high-molecular-weight complexes, allowing the formation of the ring-like structures 21 (Figure 1**B**).

PHBs have broad functions in mitochondria. They promote mitochondrial protein synthesis, maintain the structure and morphology of mitochondrial cristae 1, 27, and regulate the copy number of mitochondrial DNA (mtDNA) 27. In addition, PHBs protect newly imported proteins from degradation by the m-AAA (mitochondrial ATPases associated with diverse cellular activities) protease, act as chaperones for newly synthesized proteins of the mitochondrial complex I thereby regulating electron transport activity and reactive oxygen species (ROS) production 1, 28, 29, and regulate optic atrophy (Opa1) during mitochondrial fission and morphogenesis 26–28. Additionally, PHB1 and PHB2 serve as receptors that facilitate the incorporation of damaged mitochondria into autophagosomes, thereby regulating both Parkin-dependent (specific to PHB2) 30 and Parkin-independent Nix/Bnip3L-induced mitophagy (specific to PHB1) 31. These functions of PHB1 and PHB2 are crucial for the health and functionality of the mitochondrial pool; the deficiency of PHBs drives disease pathogenesis, including chronic inflammation, neurodegeneration, and tumorigenesis 16, 17, 32–34.

The mitochondria are essential to regulate the metabolism of macromolecules and produce energy, both crucial processes for cell survival and maintenance 35. Under normal conditions, pyruvate is oxidized via the tricarboxylic acid (TCA) cycle, generating ATP through the electron transport chain of the mitochondria via oxidative phosphorylation (OXPHOS) 35. Many pathogens remodel cellular metabolism to enhance their survival by slowing down the TCA cycle, reducing OXPHOS enzymes, and inducing aerobic glycolysis 35. These metabolic shifts often lead to reduced production of pro-inflammatory cytokines and increased pathogenic growth 35, 36. Manipulation of the host mitochondria by pathogens is an evolutionary mechanism, as they are hubs for innate immune signaling 37. Understanding how mitochondrial dynamics and energetics are affected or hijacked by pathogens is relevant to dissecting the pathogenesis of infectious diseases, and the role of PHBs in this mitochondrial-pathogen axis will be discussed in Section “Mitochondrial PHBs and Viral and Bacterial Infections”.

### Cytoplasm

The protein structural domains of PHBs support dynamic shuttling between the mitochondria, cytosol, and nucleus 38. Although the cytosol is the least characterized of these locations, PHBs have been reported to interact with proteins involved in cytoskeletal transport, cellular signaling, and cell membrane receptors and proteins 12 and will be discussed in Section “Cytoplasmic PHBs and Viral Infections”. However, more research is required to characterize whether PHBs heterodimerize in these cellular compartments 39.

## PLASMA MEMBRANE PHBS AND VIRAL AND BACTERIAL INFECTIONS

### Viral infections

Viruses are ubiquitous biological entities characterized by short sequences of nucleic acids of RNA or DNA encapsulated in a protein shell 40. Despite lacking ribosomes, vital for protein synthesis, they attack a broad spectrum of organisms and induce a wide array of diseases. Viruses exploit the host machinery to translate their messenger RNA (mRNA) into proteins, facilitating their assembly and replication within the host 41. As the most widespread and diverse pathogens on Earth, they pose significant threats to public health, agriculture and ecology 42. Therefore, it does not come as a surprise that they have caused many pandemics throughout history, including the Spanish flu, Ebola and COVID-19 43. The mechanisms by which viruses establish infection in their hosts stem from both their own genome and their ability to hijack host cellular processes, underscoring the importance of understanding these adaptive strategies. In this section, we dissect the interactions between viruses and the host proteins PHB1 and PHB2 in the plasma membrane, exploring whether these interactions elicit a viral response influencing life cycle or a host response to combat viral establishment.

#### Dengue virus (DENV)

DENV is an arthropod-borne virus comprising four serotypes (DENV-1 to DENV-4). Human infection occurs through the bite of infected *Aedes aegypti* (*A. aegypti*) and *Aedes albopictus* (*A. albopictus*) mosquitoes, prevalent in tropical regions. Female mosquitoes acquire DENV from a viraemic host during a blood meal, enabling viral replication in their tissues and salivary glands 44. Clinically, DENV infection ranges from mild dengue fever to fatal dengue shock syndrome 45, 46. DENV places nearly 40% of the global population at risk 47, 48, representing a major expanding public health threat 49. Annually, 390 million infections occur, with 3.97 billion people living in at-risk regions 50, 51. Notably, DENV-2 is of great concern due to its association with secondary infections and substantial contribution to dengue burden and mortality 52, 53.

Understanding the mechanisms of DENV entry and replication and cell surface membrane-binding proteins in mosquitoes is critical, as these processes underpin viral transmission. However, only two studies have characterized putative DENV receptor proteins in insect host cells. One identified a 45–50 kDa laminin-binding membrane protein that binds only DENV-3 and DENV-4 54. Another identified a 48 kDa cytosolic, tubulin-like protein that binds DENV-2 but is absent from the cell membrane 55.

Kuadkitkan *et al.* 56 identified the first mosquito cell surface protein capable of binding DENV-2. Using a two-dimensional Virus Overlay Binding Assay (2D-VOPBA) to separate cell membrane mixtures and probe DENV antigen preparations 57, in CCL-125 cells (*A. aegypti* cell line) and C6/36 cells (*A. albopictus* cell line), followed by mass spectrometry, they detected a 35 kDa protein corresponding to PHB2. Silencing PHB2 directly was inefficient; however, in multiple systems, loss of PHB1 leads to the concomitant loss of PHB2 due to their interdependent stability 1, 3, 26, 27, 58, independent of RNA levels 26. In siRNA-mediated knockdown of PHB1 in CCL-125 and C6/36 cells, extracellular DENV-2 production decreased by 75% and 90%, respectively. To assess PHB2-mediated DENV-2 internalization, pre-treatment with an anti-PHB2 antibody reduced both extracellular and intracellular viral production, supporting a role of PHB2 as a surface receptor for DENV-2.

**Figure 2:**
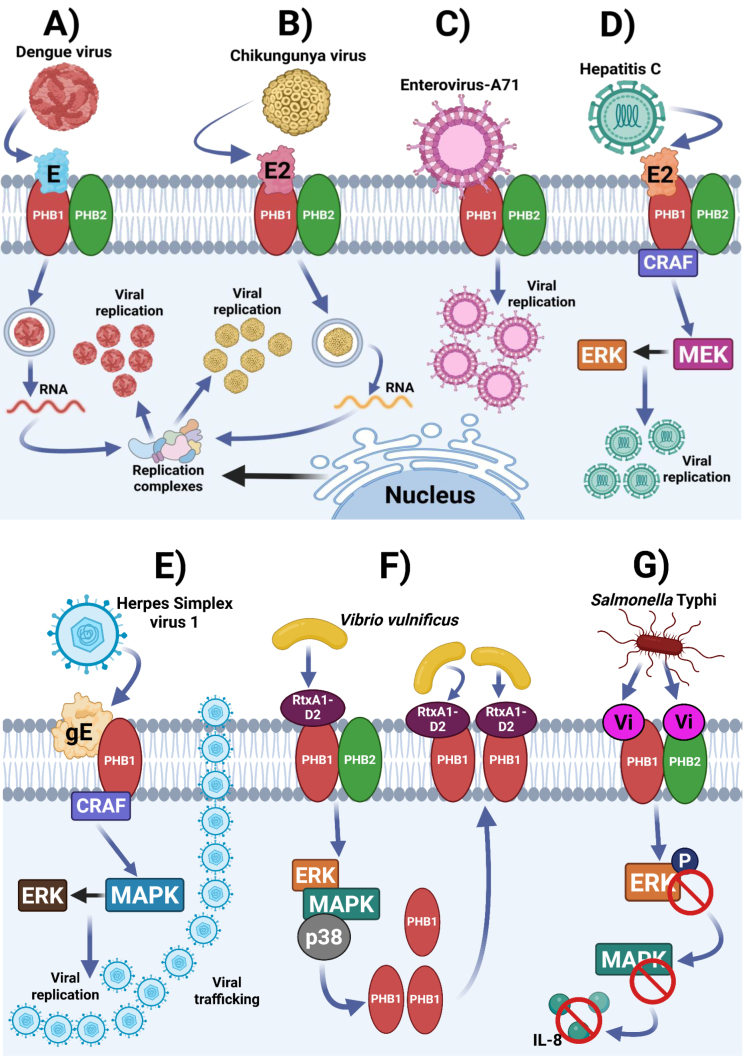
PHBs function as receptors in the plasma membrane, facilitating pathogen invasion of host cells. Subcellular localization of PHBs at the plasma membrane plays a pivotal role in bacterial and viral pathogenesis, underscoring their significance as strategic host factors in infectious disease processes. Several viruses exploit membrane-associated PHBs to facilitate host cell entry. **(A)** Dengue virus and **(B)** Chikungunya virus utilize viral surface proteins to bind to PHB1 and PHB2, respectively, promoting viral attachment and internalization at the plasma membrane. These interactions enable viruses to access host cellular machinery and establish replication complexes necessary for viral propagation. **(C)** Although the precise molecular mechanism underlying Enterovirus-A71 interaction with PHB1 remains unclear, evidence indicates that PHB1 binding enhances viral entry and supports host invasion. **(D)** Hepatitis C targets PHB1 to facilitate binding and recruitment of CRAF, leading to activation of the MEK-ERK signaling cascade and enhancing viral replication. **(E)** During Herpes Simplex virus 1, the viral protein gE binds to PHB1 to recruit CRAF and activate the ERK-MAPK signaling. This binding favors the viral replication, plaque size and viral trafficking to cell surfaces or junctional complexes. **(F)** PHB1 is a critical host receptor during *Vibrio vulnificus* infection, where interaction with the bacterial protein RtxA1-D2 activates ERK-MAPK-p38 signaling. This signaling increases PHB1 expression and its colocalization to the plasma membrane, amplifying bacterial invasion efficiency. **(G)** Both PHB1 and PHB2 are molecular targets for *Salmonella*Typhiinfection. The bacterial protein Vi binds PHBs and suppresses ERK phosphorylation. As a result, the MAPK pathway activation and IL-8 production are inhibited. This mechanism highlights how the bacterium manipulates PHB-mediated signaling networks to evade host immune responses. Created in BioRender (Rivera, G. (2026) https://BioRender.com/xl2arn5).

DENV-2 entry is mediated by the viral envelope (E) glycoprotein, which contains components for host cell binding and fusion, promoting viral entry and receptor-mediated endocytosis 59, 60. Kuadkitkan *et al.* showed colocalization of the E protein with PHB1 and PHB2, and confirmed direct interaction between the E protein and PHB2 only by co-immunoprecipitation 56. PHB2 was identified as a receptor involved in DENV-2 binding and entry in mosquito cells. This observation aligns with a previous work reporting four DENV-binding proteins (77, 58, 54, and 37 kDa) in *A. aegypti* salivary gland extracts 61, where the 37 kDa protein may correspond to PHB2; however, its identity was not confirmed (Figure 2**A**).

Several studies have reported localization of PHB1 and PHB2 on the surface of mammalian cells 5, 7, 18. Given the 75% sequence homology between *A. aegypti* and human PHBs 62, a strong evolutionary conservation, PHB2 may also function as a DENV-2 surface receptor in mammalian cells. It requires further validation, but PHBs represent a promising target for therapeutic intervention against DENV, which currently lacks specific treatment or cure

#### Chikungunya virus (CHIKV)

CHIKV is a mosquito-borne arthritogenic arbovirus that infects both animals and humans 63, 64. Transmission occurs mainly through the bite of infected *A.aegypti* and *A. albopictus* 65. CHIKV infection causes Chikungunya fever, characterized by high fever, headache, myalgia, rash, and prominent polyarthralgia 66. Viral entry into host cells occurs via clathrin-mediated endocytosis 67 and micropinocytosis 68. It has broad cellular tropism, including dendritic cells, macrophages, endothelial cells and myocytes, contributing to joint pathology and erosive disease in patients with chronic arthritis 69, 70. Over the past decade, four million CHIKV cases were reported in the Americas 71. Rising temperatures, increased rainfall, and humidity are expanding mosquito habitats and accelerating CHIKV transmission.

Wintachai *et al.* identified PHB1 as a CHIKV receptor in human cells 72. Using CHME-5 human microglial cells, relevant to the neurological manifestations of CHIKV infection 73, 74, PHB1 was detected by 2D VOPBA and mass spectrometry. Neutralizing anti-PHB1 antibodies caused a dose-dependent decrease in CHIKV infection and viral production, implicating PHB1 in viral entry. E proteins mediate CHIKV entry, with E2 facilitating receptor binding and E1 driving pH-dependent membrane fusion 75. PHB1 colocalized with E2 on the cell surface, and PHB1 silencing reduced infection by 30%, supporting its role as a surface binding protein. However, incomplete inhibition following antibody neutralization or silencing suggests that additional entry factors contribute to CHIKV internalization.

Only IXCHIQ (VLA1553), a live-attenuated vaccine authorized for individuals ≥ 12 years old, is the only FDA (Food and Drug Administration)-approved CHIKV vaccine 76. It elicits durable homotypic and heterotypic neutralizing responses against three CHIKV strains 77, 78, yet the virus mutation rate ( ∼ 2.8%) poses a risk of vaccine escape 79. For example, a valine substitution in the E protein caused a more infectious variant linked to global outbreaks since 2004 78, 80. Thus, identifying host receptors involved in CHIKV entry is critical for developing more effective and durable antiviral strategies.

Consistent with this approach, Wintachai *et al.* 81 evaluated flavaglines, natural compounds with known anticancer, neuroprotective, and cardioprotective properties 82–84, for their ability to interfere with PHB1–CHIKV binding. In HEK293T/17 cells (human embryonic kidney tissue cell line), two synthetic flavaglines (FL3 and FL23) inhibited CHIKV infection and viral production by 50%, while reducing PHB1–CHIKV colocalization. Since the compounds were applied before infection, their primary effect likely occurs at the viral entry stage, with minimal post-entry activity 81. These findings suggest that PHB1-targeting ligands, such as flavaglines, may represent promising prophylactic strategies to block CHIKV entry and support the development of antivirals less susceptible to viral mutation (Figure 2**B**).

#### Enterovirus-A71 (EV-A71)

EV-A71 is a human-specific virus 85 primarily transmitted via the fecal–oral route 86, though respiratory secretions also contribute to person-to-person spread 87. Early viral replication occurs in the Peyer’s patches of the small intestine, which are lymphoid tissues that act as the immune system’s first line of defense against pathogens and antigens 88, and the lymphoid tissues of the tonsillar crypts 89, 90. EV-A71 mainly affects children ≤ 5 years old, with the highest severity observed in those ≤ 3 years old, while adult cases are rare 91. Most pediatric cases are self-limiting and present as hand-foot-and-mouth disease or herpangina. However, severe neurological complications occur, with a mortality rate of up to 0.03% 92.

Despite well-characterized mild clinical manifestations, the neuropathogenesis of EV-A71 remains poorly understood. Recurrent outbreaks, most notably in the Asia-Pacific region and occasionally in Europe, underscore its global relevance 93. EV-A71’s high mutation rate (10 ${}^{3}$ –10 ${}^{5}$ mutations per nucleotide per replication cycle) 94 hinders vaccine development, and effective therapeutics remain unavailable.

By proteomic profiling and MALDI-TOF mass spectrometry in EV-A71-infected NSC-34 motor neuron-like cells, Too and colleagues identified PHB1 as a top host factor linked to EV-A71 neuropathogenesis via STRING network analysis 95. Pre-treatment with anti-PHB1 antibodies resulted in a dose-dependent reduction in viral titers, indicating PHB1-mediated viral entry. Proximity ligation assay and co-immunoprecipitation confirmed physical interaction between EV-A71 and surface-expressed PHB1, supporting its role as an entry receptor (Figure 2**C**).

#### Hepatitis C virus (HCV)

HCV is a lipid-centric virus that infects humans and chimpanzees, having a strong tropism for hepatocytes 96, 97. In the bloodstream, HCV exists as free virions or complexed with host low-density lipoproteins 98 and enters hepatocytes via clathrin-mediated endocytosis. Viral entry requires numerous host proteins, including occludin, glycosaminoglycans and claudin-1 99, 100. HCV infection can be acute or chronic. While acute infection is often asymptomatic 101, 50–80% of these cases progress to chronic infection 102, which can lead to fibrosis, cirrhosis, hepatocellular carcinoma, and death.

HCV transmission occurs mainly via percutaneous exposure to infected blood 103 with vertical transmission as a secondary route from mother to infant. In the United States, HCV has a prevalence of 62–79 million individuals 104. Current treatment relies on direct-acting antivirals (DAAs), making HCV a curable chronic infection 105. Nevertheless, the high treatment cost (up to $84,000 per 12-week course in the U.S.) limits their effectiveness and accessibility 106, 107. Hence, affordable novel host-targeted antivirals are a priority.

Liu and colleagues identified PHB1 and PHB2 as key interactors of the HCV E2 protein in infected human hepatoma cells (Huh7.5.1) 108. Proteomics and co-immunoprecipitation confirmed direct PHB1 and PHB2 binding with E2, independent of other viral components. siRNA-mediated knockdown of PHB1 and PHB2 in Huh7.5.1 cells and primary human hepatocytes blocked viral entry and reduced HCV RNA levels without affecting viral attachment, indicating a role in post-binding membrane fusion 108. Confocal microscopy and biotinylated localized PHB1 and PHB2 to the plasma membrane of Huh7.5.1 cells 108.

PHB1 and PHB2 interact with EGFR and c-Raf (CRAF), components of a signaling axis critical for HCV entry 108. EGFR is a cell-surface receptor considered a cofactor to HCV entry and infection 9. EGFR-dependent signaling promotes CD81–claudin–1 complex formation 99, 100, essential for HCV viral entry. CRAF, plays a role in cell growth, differentiation, and other processes, and PHB1 recruits it to the plasma membrane 109. Disruption of the PHB1-CRAF axis via CRAF silencing 110 of the PHB1 C-terminal domain abrogates HCV infection (Figure 2**D**).

Rocaglamide A (Roc-A), which disrupts PHBs-CRAF interactions in leukemic models 111, blocked the PHB-CRAF-MEK-ERK signaling pathway, a cascade exploited by HCV 108. In addition, it restored interferon-stimulated genes (ISGs), inhibited interferon (IFN) expression, and reduced PHB1, PHB2 and CRAF expression on cells 108. Together, these findings highlight PHBs and the PHBs-CRAF axis as promising host-targeted antiviral strategies.

#### Herpes Simplex virus 1 (HSV-1)

HSV-1 is a ubiquitous human pathogen capable of infecting multiple cell types 112. Commonly, it causes lesions of the lips, eyes, mouth, and genitals, while severe complications such as encephalitis and herpes keratitis can occur in neonates and immunocompromised individuals 113. Infections with HSV-1 are of high importance since they significantly increase the risk of acquired immunodeficiency syndrome (AIDS) 114. Although antivirals such as acyclovir and foscarnet are used to manage infection, they do not eliminate the virus 114. Therefore, a deeper understanding of HSV-1 replication and establishment is key to developing novel treatments.

Envelope glycoprotein E (gE) is essential for HSV-1 cell-to-cell spread, directing enveloped virions to the junctional cell surface 115, 116. Using tandem affinity purification and mass spectrometry (MS)-based proteomics, Watanabe and colleagues identified PHB1 as a gE-interacting host protein 117. PHB1 overexpression in HeLa cells increased plaque size, whereas genetic or pharmacological depletion (Roc-A) reduced plaque formation 117. Notably, gE knockout did not further decrease plaque size in PHB1-deficient cells, confirming that PHB1 promotes HSV-1 cell-to-cell spread in a gE-dependent manner 117. Reduced PHB1 expression was shown to cause cytoplasmic accumulation of virions and decreased extracellular release, indicating impaired transport of enveloped particles to the cell surface 117, 118. As a scaffold protein in the MAPK/ERK pathway 119, PHB1 is required for efficient viral spread. Pharmacological inhibition of PHB1 or MAPK/ERK signaling reduced plaque size and led to virion accumulation, confirming that PHB1 promotes gE-dependent cell-to-cell spread via the MAPK/ERK signaling pathway 117. Although the precise mechanism of gE-PHB1 interaction is unclear, gE localizes to the plasma membrane in infected cells and may recruit PHB1 to activate MAPK/ERK signaling 116 (Figure 2**E**). This could facilitate Raf recruitment to promote virion trafficking to the cell surface or junctional membranes 116, 117.

### Bacterial infections

Bacteria are ubiquitous, single-celled organisms lacking a nuclear membrane and capable of surviving extreme environmental conditions 120. Unlike viruses, bacteria possess their own replication machinery. Typically, bacteria have few chromosomes with a defined origin of replication 121. Bacterial classification commonly relies on Gram staining, which reflects differences in cell envelope structure: Gram-positive bacteria have thick peptidoglycan layers (20–80nm) 122, while Gram-negative bacteria possess thinner walls (<10nm) and an outer membrane with porins 122, 123. These structural differences influence bacterial resistance to environmental stressors, including antibiotics. In 2019, 7.7 million deaths worldwide were found to be linked to infections caused by 33 bacterial pathogens, accounting for 13.6% of global deaths that year 124. Escalating antibiotic resistance—driven by diverse adaptive mechanisms—has intensified the global health threat posed by bacterial pathogens 125.

Bacteria employ diverse mechanisms to invade host cells and establish infection, yet host defense strategies against their virulence factors remain incompletely understood. This section explores the interactions between bacterial pathogens and the proteins PHB1 and PHB2, highlighting their emerging roles in infection biology and their potential as alternative therapeutic targets to mitigate bacterial pathogenesis and antibiotic resistance.

#### *Vibrio vulnificus* (*V. vulnificus*)

*V. vulnificus* is a Gram-negative, waterborne and foodborne pathogen 126 transmitted through consumption of contaminated seafood or exposure of open wounds to contaminated saltwater or brackish water 127. Clinically, infection presents as gastroenteritis, septicemia or wound infection 126. In the United States, 150–200 *V. vulnificus* cases are reported annually, with a 20% mortality rate, often within 48 hours of symptom onset 128. Its prevalence is projected to rise with climate change and the expansion of saline waters 129, 130.

A hallmark of *V. vulnificus* is its potent cytotoxicity toward eukaryotic cells 131, mediated by key virulent factors, including cytolytic hemolysin, elastolytic protease and RTX (RtxA1) 132. RtxA1 mediates host cell death, cytoskeletal disruption, hemolysis, and tissue invasion 132–134. While largely homologous to *V. cholerae* RTX, *V. vulnificus* harbors a unique domain (RtxA1-D2) with no significant similarity to known proteins 135, 136. Expression of RtxA1-D2 in HeLa cells (immortalized human epithelial cells derived from cervical cancer) induced plasma membrane ballooning, implicating a distinct mechanism of cytotoxicity 132.

A yeast two-hybrid screen identified PHB1 as a host-interacting protein of the RtxA1-D2 domain 137, a finding confirmed by co-immunoprecipitation in HeLa cells infected with *V. vulnificus* 137. Wild-type, but not RtxA1-deficient strains, induced time-dependent upregulation of PHB1 at the plasma membrane with no effect on PHB2. Pharmacological inhibition of ERK and p38 MAPK pathways suppressed this effect, linking MAPK signaling to PHB1 expression 133. Notably, PHB1 knockdown or antibody-mediated neutralization significantly reduced RtxA1-induced cytotoxicity 137 (Figure 2**F**).

These findings suggest that PHB1 contributes to a positive feedback loop amplifying RtxA1-mediated cytotoxicity by binding to the RtxA1-D2 domain and activating the p38/ERK MAPK signaling. However, whether surface-localized PHB1 serves as a receptor for RtxA1 internalization is unclear. Further mechanistic studies are required to define the role of PHB1 in *V. vulnificus* entry and invasion. Given the rising resistance to frontline antibiotics such as penicillin (68%) and ampicillin (53%) 138, elucidating host-pathogen interactions may inform alternative therapeutic strategies to address antibiotic resistance.

#### *Salmonella*Typhi (*S.*Typhi)

*Salmonella enterica* serovar Typhi (*S.*Typhi) is a human-restricted, Gram-negative intracellular pathogen that causes typhoid fever, transmitted via the fecal–oral route 139, 140. Typhoid fever affects 11–21 million individuals annually, with ∼ 230,000 deaths 141, predominantly in regions with limited sanitation and clean water 142. Clinical manifestations include persistent high fever, abdominal pain, and gastrointestinal disturbances 139. The rise of multidrug-resistant strains to chloramphenicol and ampicillin 143, coupled with the limited efficacy of existing vaccines 144, has fueled the spread. Therefore, a better understanding of host-pathogen interactions underlying invasion and systemic dissemination is critical to develop effective treatments 140.

The Vi outer capsular polysaccharide is a key virulence factor that protects *S.*Typhifrom phagocytosis and complement-mediated lysis 145, and enhances bacterial survival in macrophages by suppressing autophagy 146. However, its interaction with intestinal epithelial cells (IECs), the primary targets of infection, has only recently been characterized.

The Vi antigen binds to human IECs in a dose-dependent manner 147. By immunoprecipitation and mass spectrometry, PHB1 and PHB2 were identified as Vi-interacting membrane proteins, localized in lipid rafts 147. Given the role of lipid rafts in signal transduction 148, Vi-PHBs’ association suggests a role in modulating host signaling. Indeed, Vi exposure reduces IL-8 production in epithelial and immune cells and it is associated with diminished neutrophil infiltration, highlighting a potential immunomodulatory role of PHBs during *S.*Typhiinfection 149.

Vi reduces ERK phosphorylation, implicating inhibition of the MAPK pathway in the dampening of early immune responses 147. Vi targeting membrane-associated PHB1 150 impairs the MAPK signaling and PHB1 redistribution within lipid rafts in IECs, monocytes, and T cells (Figure 2**G**). This suggests a strategy by *S.*Typhi to evade immune detection during invasion 151, and identifies PHB1 as a modulator of Vi-induced immune suppression.

## MITOCHONDRIAL PHBS AND VIRAL AND BACTERIAL INFECTIONS

### Viral infections

#### Enterovirus-A71 (EV-A71)

Besides the EV-A71-PHB1 interaction at the cell surface, mitochondrial PHB1 plays a role in the infection cycle of this virus. EV-A71 RNA was transfected into NSC-34 PHB1-depleted cells to bypass viral entry 95. This resulted in significantly reduced viral titers, which indicates involvement of PHB1 in intracellular replication. Immunostaining further revealed colocalization of PHB1 with EV-A71 capsid and non-structural proteins in the mitochondria, reinforcing its dual role in viral entry and replication 95.

**Figure 3:**
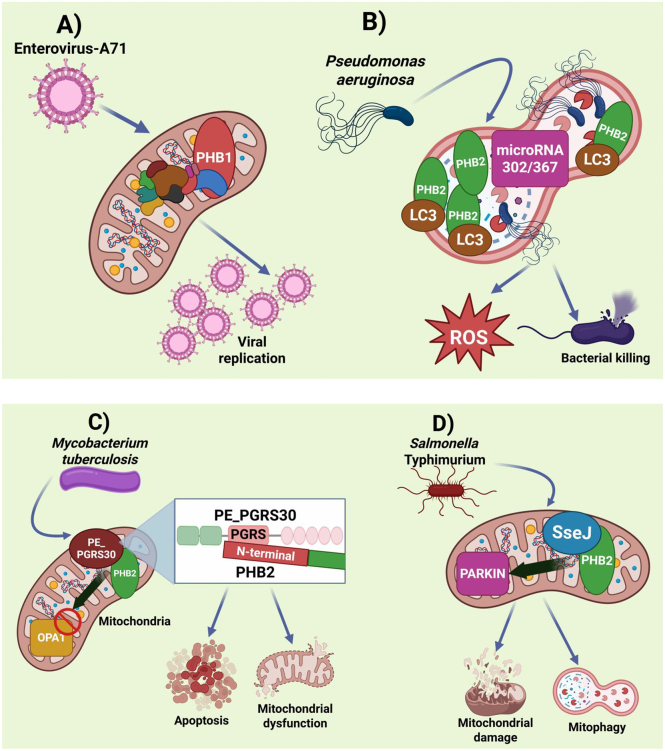
Exploitation of PHBs in mitochondria by viruses and bacteria can lead to mitochondrial damage. Subcellular localization of PHBs in mitochondria offers valuable insights into their functional roles in cellular homeostasis and immune regulation, highlighting their potential as promising therapeutics for control of infectious diseases. **(A)** PHB1 functions as a critical host factor for Enterovirus-A71, facilitating viral replication through the recruitment of host-derived replication complexes. **(B)***Pseudomonas aeruginosa* is controlled by the host cell through an interaction between microRNA-302/307 and PHB2, increasing ROS production and subsequent bacterial killing. **(C)***Mycobacterium tuberculosis* and **(D)***Salmonella* Typhimurium use their bacterial effectors to interact with PHB2. *Mycobacterium tuberculosis* uses the PGRS domain from its virulence factor PE_PGRS30 to bind to the N-terminal domain of PHB2. This disrupts PHB2 function and inhibits OPA1 from being processed. *Salmonella* Typhimurium uses its bacterial effector SseJ to bind PHB2; this interaction activates PARKIN, an E3 ubiquitin ligase. With both pathogens, interactions with PHB2 lead to mitochondrial damage and dysfunction, and autophagy. As a result, infection and dissemination are enhanced. Created in BioRender (Rivera, G. (2026) https://BioRender.com/c0ebwr2).

EV-A71 exploits mitochondria in NSC-34 cells as replication platforms, with co-localization of PHB1, dsRNA, and mitochondrial markers indicating PHB1’s association with the viral replication complex. Although enteroviruses use the endoplasmic reticulum (ER) as the main replication site 152, mitochondrial fractions from infected NSC-34 cells contained viral capsid proteins (VP1, 3D, 3CD) and lacked the ER marker calreticulin 95. Consistent with this, VP1 has also been detected in the mitochondria of HeLa cells 153. Transmission electron microscopy further revealed mitochondrial clustering around replication complexes. These findings implicate mitochondrial PHB1 as a critical host factor co-opted by EV-A71 to facilitate replication (Figure 3**A**).

### Bacterial infections

#### *Pseudomonas aeruginosa* (*P. aeruginosa*)

*P. aeruginosa* is an aerobic Gram-negative opportunistic bacterium with a high genetic capability to grow and tolerate a wide variety of environments 154. It infects patients with cystic fibrosis, burn wounds, chronic obstructive pulmonary disorder, immunodeficiency, and severe infection requiring ventilation 155. It accounts for 7% of healthcare-associated infections 156. This pathogen exhibits intrinsic antibiotic resistance (low cell permeability, multiple efflux pumps, and antibiotic-modifying enzymes), can evolve antibiotic resistance through mutational changes, and acquire resistance genes through horizontal transfer 157.

Huang and colleagues 158 identified the role of the microRNA-302/367 cluster in regulating mitophagy host defense against *P. aeruginosa* in macrophages. Emerging evidence suggests microRNAs regulate autophagy-related genes and inflammatory responses 159, 160. The microRNA-302/367 cluster upregulated PHB2 expression 158, and confocal microscopy confirmed PHB2 recruitment during mitophagy in *P. aeruginosa* infection. PHB2 knockdown significantly reduced mitophagy and bacterial clearance driven by microRNA-302/367 158. Consistently, PHB2-depleted macrophages failed to clear *P. aeruginosa*. These findings establish PHB2 as a critical mitophagy receptor whose upregulation in response to microRNA-302/367 activation is pivotal for mitophagy induction in macrophages. PHB2 facilitates ROS production and promotes bacterial clearance. Although the precise mechanisms by which PHB2 may interact with *P. aeruginosa* remain unclear, these findings link microRNA-mediated regulation of mitophagy for bacterial clearance and highlight a synergistic role for microRNA-302/367 and PHB2 in enhancing host defense (Figure 3**B**).

#### *Mycobacterium tuberculosis* (Mtb)

Mtb, the causative agent of tuberculosis (TB), is an intracellular respiratory pathogen with a lipid-rich, mycolic acid-containing outer membrane that confers low permeability 161, 162. Humans are the sole reservoir, with transmission occurring primarily via aerosolized droplets 163. Infection outcomes depend on host response: latent TB is asymptomatic and non-transmissible; whereas active TB manifests with prolonged cough, hemoptysis, fatigue, fever, and requires immediate treatment 164, 165. In 2023, TB caused 10.8 million cases and 1.25 million deaths globally 166, 167, remaining the leading cause of death among individuals with HIV. The economic burden is substantial, with the United States spending $22 billion annually on prevention, diagnosis, and treatment 166. Current therapy involves a prolonged, costly four-drug regimen (isoniazid, pyrazinamide, rifampicin, and ethambutol), often leading to poor adherence 168. The rise of multidrug-resistant Mtb strains underscores the urgency of understanding host–pathogen interactions to develop durable therapeutic strategies.

PE_PGRS30, a virulence factor of Mtb, contains a highly conserved Pro-Glu (PE) domain, a polymorphic GC-rich sequence (PGRS) domain, and a C-terminal (CT) domain (148). Iantomasi *et al.* demonstrated that deletion of PE_PGRS30 impairs Mtb’s ability to inhibit phagosome–lysosome fusion, reducing lung colonization and tissue damage in murine and human macrophages 169. While the CT domain is dispensable for virulence, expression of PE_PGRS30, but not related family members, induces macrophage cell death and inhibits proliferation 170, implicating the PE and PGRS domains in cytotoxicity and host-pathogen interaction. Mechanistically, PE_PGRS30 binds to PHB2 170, specifically to its N-terminal mitochondrial targeting sequence 170, disrupting PHB2 function. This interaction preserves OPA1 from proteolytic processing, leading to mitochondrial dysfunction and apoptosis. These findings suggest that PE_PGRS30 hijacks host mitochondrial pathways via PHB2 to promote macrophage death and dampen host immune responses during infection (Figure 3**C**).

#### *Salmonellaenterica* serovar Typhimurium *(S.*Typhimurium*)*

*S.* Typhimurium is a Gram-negative, facultative intracellular pathogen with a broad host range and a high metabolic versatility 171. It persists across diverse environments and is primarily transmitted via ingestion of contaminated food or water 171, 172. Upon oral acquisition, *S.* Typhimurium colonizes the large intestine, where replication occurs 172. Clinical symptoms typically arise within 12–72 hours and include diarrhea, fever, abdominal cramps and nausea 173. Although generally self-limiting, infection in immunocompromised patients can be severe or fatal if not treated promptly with antibiotics 173. *S.* Typhimurium is a significant public health burden due to frequent foodborne outbreaks 174–176, prolonged hospitalizations 177, and rising healthcare costs 178. Antibiotic-resistant strains further complicate treatment 179, 180, underscoring the need for alternative therapeutics targeting mechanisms of invasion, intracellular survival, and immune evasion.

A recent study showed that *S*. Typhimurium infection in RAW264.7 macrophages triggers severe mitochondrial damage and subsequent mitophagy 181. The T3SS-2 effector Secretion system effector J (SseJ) was essential for *S*. Typhimurium-induced mitochondrial damage. SseJ interacts with PHB2 to induce mitophagy in the host cell via the Parkin-dependent pathway, facilitating bacterial intracellular proliferation 181. *In vivo,* mice treated with Roc-A (PHBs inhibition) and Mdivi-1 (mitophagy inhibition) have reduced bacterial pathogenicity. Importantly, infection with *S*. Typhimurium- Δ *SseJ* exhibited lower systemic infection 181. Therefore, PHB2 is identified as a mitochondrial target of *S*. Typhimurium, presenting the SseJ-PHB2-mitophagy pathway as a novel potential therapeutic target (Figure 3**D**).

## CYTOPLASMIC PHBS AND VIRAL INFECTIONS

### Viral infections

#### Influenza A virus subtype H1N1 (H1N1)

The H1N1 influenza virus expresses hemagglutinin (HA), a major viral antigen responsible for eliciting neutralizing antibody response 182. Monoclonal antibodies targeting HA have been developed for vaccine-related applications, including the widely used H1-50 antibody. However, H1-50 exhibits cross-reactivity with PHB1 in pancreatic islet β -cells 183, 184. This interaction is mediated by epitope spatial conformation, with H1-50-PHB1 binding to the spatial protein structure within the host cytoplasm 183, 184.

**Figure 4:**
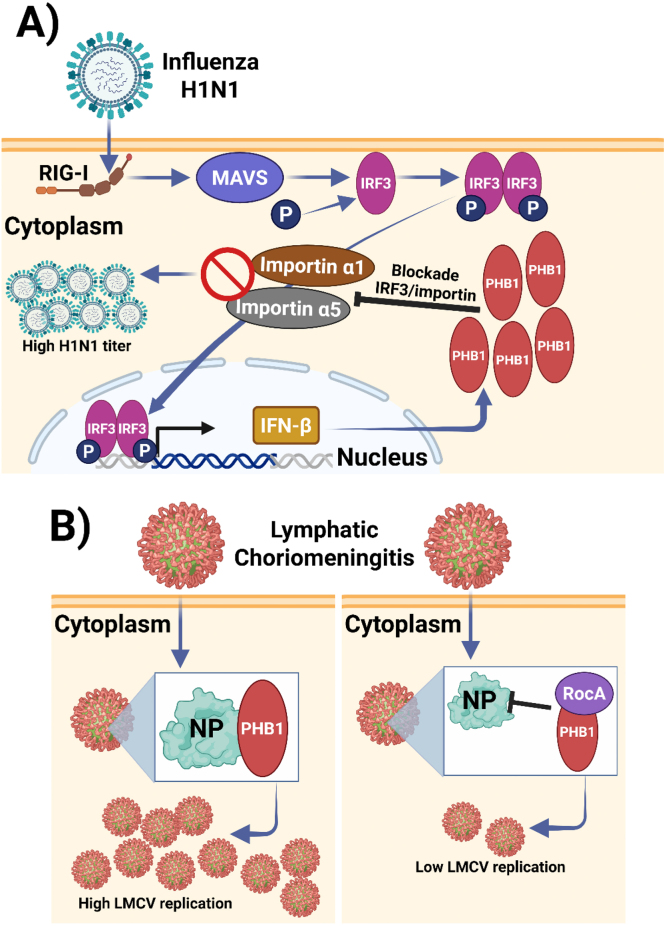
PHBs in the cytoplasm are hijacked by viruses to allow more efficient viral replication. Cytoplasmic localization of PHB1 facilitates viral replication. **(A)** In the context of influenza H1N1 infection, PHB1 interacts with viral components to disrupt the nuclear localization of IRF3. This impairment inhibits IFN- β transcriptional activation, suppressing the host antiviral response and promoting viral replication. **(B)** Similarly, lymphatic choriomeningitis exploits PHB1 by interacting with its nucleoprotein (NP), which supports efficient viral replication in host cells. Pharmacological inhibition of PHB1 with Rocaglamide A disrupts the NP–PHB1 interaction, leading to reduced viral replication. These findings collectively highlight the critical role of PHB1 in facilitating efficient viral replication. Created in BioRender (Rivera, G. (2026) https://BioRender.com/0gq266h).

Zou and colleagues first identified a role for PHB1 in H1N1-triggered innate immune response 185. PHB1 silencing reduced viral titers, whereas PHB1 overexpression produced the opposite effect. During viral infection, the RIG-I signaling pathway activates mitochondrial antiviral signaling protein (MAVS), leading to interferon regulatory factor 3 (IRF3) activation and formation of IRF3/IRF3 or IRF3/IRF7 complexes to translocate to the nucleus to induce interferon β (IFN- β ) transcription to clear the infection 185. H1N1-induced IFN- β signaling increased PHB1 expression at both mRNA and protein levels. PHB1 overexpression suppressed IFN- β production, suggesting a negative regulatory role in antiviral signaling 185. Mechanistically, PHB1 interacts with the nuclear localization signal (NLS) of IRF3, disrupting the IRF3 binding to importins α 5/ α 7 and impairing nuclear accumulation without fully blocking translocation 185. The attenuation of IRF3 nuclear localization reduces IFN- β production, thereby facilitating efficient H1N1 infection (Figure 4**A**).

#### Lymphocytic Choriomeningitis virus (LCMV)

LCMV is a rodent-borne virus that can infect humans after contact with rodent secretions or excreta by inhalation or aerosolized particles 186. Human-to-human transmission can occur during intrauterine development or organ transplantation 187. Although healthy individuals suffer asymptomatic or mild infections of LCMV, immunocompromised patients can develop life-threatening conditions such as meningitis or encephalitis 187. A detailed understanding of how LCMV interacts with the host is needed since there is no antiviral treatment. Current treatment offers supportive care to alleviate the symptoms.

In LCMV, the nucleoprotein (NP) is the most abundant viral protein in infected cells and virions 188. NP has a key role in RNA synthesis and exhibits an anti-IFN activity via IRF3 blockade 188. PHB1 was identified as a pro-viral host factor interacting with NP by Iwasaki and colleagues 189. By using Roc-A, a PHB1 inhibitor, in A549 cells infected with LCMV, they observed dose-dependent inhibition of LCMV multiplication 189 (Figure 4**B**). Roc-A did not inhibit LCMV cell entry, but it inhibited LCMV replication, gene transcription and budding efficiency 189.

#### Theiler’s mouse encephalomyelitis virus (TMEV)

TMEV is a recurring enteropathogen in mice, often used in two viral infection mouse models for human diseases such as multiple sclerosis and epilepsy 190. Depending on the mouse strain, the disease development varies. The C57BL/6J mouse strain develops acute seizures that progress into epilepsy 191.

Alipoprotein L9a and L9b (*Apol9a* and *Apol9b*) are isoforms, cataloged as interferon-stimulated genes (ISG) with antiviral activity 192. Both are constitutively expressed in murine liver, pancreas and adipose tissue, and their expression can be further upregulated by type I IFN, which acts as a first-line innate immune defense against viruses, bacteria, and parasites 192.

**Figure 5:**
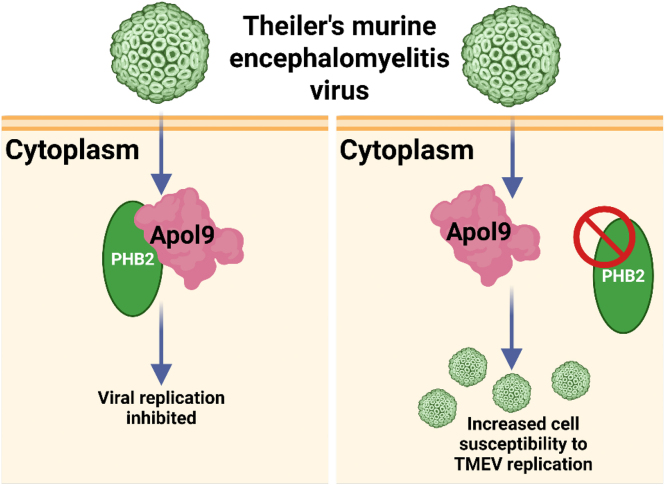
PHBs contribute to controlling Theiler virus infection in the cytoplasm. During Theiler’s murine encephalomyelitis virus infection, cytoplasmic localization of PHBs contributes to inhibiting viral replication in the hot cells. Host protein Apol9 interacts with the PHB complex to mitigate Theiler virus replication. Genetic downregulation of PHB2 abrogates the antiviral activity of Apol9 and increases viral replication. This anti-viral activity of PHB2 against Theiler virus differs from the pro-viral role of PHBs with the viruses previously discussed. Further studies targeting PHBs-Apol9 interaction are needed to elucidate the antiviral effect exerted by this complex and its potential application to other pathogens. Created in BioRender (Rivera, G. (2026) https://BioRender.com/y0a51do).

Apol9 is localized in the cell cytoplasm and it inhibits the replication of TMEV. By co-immunoprecipitation, it was discovered that Apol9 interacts with PHBs to exert antiviral activity 192, unlike their role in other viruses, where they contribute to viral infection. The mechanisms of how this interaction happens were not addressed, but it was established that the interaction occurs in the cytoplasm. Although the knockdown of PHB2 did not abrogate the antiviral activity of Apol9a and Apol9b, it exhibited increased cell susceptibility to TMEV, suggesting that PHBs may act as chaperones to stimulate the antiviral activity of Apol9 192. More studies are required to understand how, as opposed to different viruses, PHBs work with Apol9 to restrict viral replication (Figure 5). Establishment of a mechanistic effect on this Apol9-PHBs interaction may elucidate new therapies potentially worth exploring for other viruses to control infection.

## THERAPEUTIC TARGETING OF PHBS AND FUTURE DIRECTIONS

Given the role of PHB1 and PHB2 in viral and bacterial infections, PHBs provide potential targets for anti-infectious therapies. Synthetic flavaglines, such as FL3 and FL23, have been shown to bind PHBs and protect against inflammation, tumorigenesis, and toxicity in various cell types 83, 193–195. The anti-infectious potential of flavaglines was demonstrated in *in vitro* studies in which FL3 and FL23 efficiently prevented CHIKV infection in microglial cells 81. Furthermore, Roc-A, as an established inhibitor of PHBs, effectively blocked plasma membrane-localized PHBs and decreased HCV infection 111 or decreased mitochondria-associated PHB1 and inhibited neuropathogenesis during EV-A71 infection 95. Access of PHBs in the cytoplasm by LCMV and influenza H1N1 influences viral replication and inhibition of IFN- β production to control viral infection, respectively. These studies suggest benefits of altering PHBs located at the plasma membrane and cytoplasm involved in microbe entry into the host cell, as well as PHBs located at the mitochondria associated with microbe replication and host mitochondrial integrity. Such targeting of host PHBs is especially important when considering vaccines designed against viruses or bacteria that can exhibit rapid mutation rates and escape vaccine-induced immunity over time.

### PHBs beyond pathogenic interactions

Beyond the role of PHBs as host factors that interact with bacteria and viruses, emerging evidence suggests that PHBs may also interact with viral proteins in ways that can be therapeutically exploited. For example, adult T-cell leukemia (ATL), an aggressive malignancy caused by infection with human T-cell lymphotropic virus 1 (HTLV-1), remains difficult to treat effectively 196. To explore potential therapeutic mechanisms, researchers introduced the Vpr protein from HIV-1 into HTLV-1-transformed C8166 cells to evaluate its cytotoxicity effects 197. Vpr transduction induced several hallmarks of apoptosis, including G2/M cell-cycle arrest, mitochondrial membrane potential loss, nucleic acid condensation and activation of caspase 3 and 7. These findings demonstrated that apoptosis is the primary mechanism underlying Vpr-mediated C8166 cell death 197 and suggested that mitochondrial dysfunction contributes to this process. Notably, Vpr expression was associated with downregulation of PHB1 197. These observations highlight a potential link between PHB1 and virus-mediated apoptotic pathways, suggesting that viral proteins can be leveraged to probe PHB1-dependent mechanisms beyond pathogen entry. A deeper understanding of the molecular interactions between PHB1 and Vpr may therefore provide insight into strategies for targeting ATL cells, potentially through apoptosis-inducing and anti-inflammatory mechanisms.

New evidence also suggests that PHBs may have therapeutic potential in metabolic diseases such as obesity. Won and colleagues developed an adipocyte-targeting fusion-oligopeptide gene carrier consisting of an adipocyte targeting sequence and 9-arginine (ATS-9R), designed to selectively transfect to mature adipocytes 198. Notably, this construct was found to bind to PHB1. Its targeting capacity was validated through binding to fat vasculature, cellular internalization and gene expression in adipocytes. Using this system, the authors delivered a short-hairpin RNA (shRNA) to silence fatty acid binding protein 4 (shFABP4), a key lipid chaperone in fatty acid uptake and lipid storage adipocytes. In obese mice, administration of ATS-9R carrying shFABP4 resulted in metabolic recovery and reduced body weight, indicating metabolic recovery, and a new alternative to treat obesity and obesity-induced metabolic syndrome 198. These findings shed light on PHB1 as a potential molecular target for adipose-specific delivery strategies and further support the exploration of PHBs as therapeutic entry points for diseases currently lacking effective treatments.

(+)-(S)-Bakuchiol (Bakuchiol) is a phenolid isoprenoid with a chiral tetra-alkylated quaternary center that prevents mitochondrial lipid peroxidation, protects other enzymes from oxidative stress and activates the nuclear factor erythroid 2-related factor 2 (Nrf2) signaling pathway to protect against oxidative stress by ROS production 199, 200. PHB1 and PHB2 bind to Bakuchiol in the mitochondria 200. Reduction of PHB1 expression with co-treatment with Bakuchiol in cells infected with influenza H1N1 suppresses viral replication 200. The anti-influenza activity exerted by Bakuchiol is hypothesized to be due to activation of Nrf2 and the antioxidant response via oxidative stress of ROS production by targeting PHBs in the mitochondria. Although mechanistic studies are needed to understand this anti-influenza activity, bakuchiol and PHBs represent promising antiviral candidates for new therapies.

### The role of PHBs in the activation of inflammasomes

The inflammasomes are cytosolic multiprotein complexes that sense cellular stress or infection and activate caspase 1, triggering the maturation of pro-inflammatory cytokines and initiating innate immune responses 201, 202. Activation of the inflammasomes is mediated by pattern-recognition receptors (PRRs), which detect pathogen-associated molecular patterns (PAMPs) and danger-associated molecular patterns (DAMPs) 202. Among PRRs, nucleotide-binding and leucine-rich repeat receptors (NLRs) are key sensors of intracellular PAMPs and DAMPs 203 and are classified into three main subfamilies: NODs, IPAF and NLRP 203. Activation of NLRP inflammasomes results in caspase-mediated cleavage of pro-IL-18 and pro-IL-1 β into their mature forms, IL-18 and IL-1 β , as well as cleavage of gasdermin D 203. Together, this promotes inflammatory responses that help the host counteract cellular stress or infection. Given the importance of NLRP inflammasomes, several studies have investigated how interactions with host proteins modulate their activity, identifying PHBs as proteins of potential interest.

*Neospora caninum* (*N. caninum*) is an intracellular protozoan parasite that causes neosporosis, a disease associated with abortion in cattle 204. During infection, *N. caninum* secretes dense granule protein 7 (NcGRA7), which interacts with the host mitochondria and contributes to parasite invasion and modulation of host immune responses. Notably, NcGRA7 has been shown to interact with PHBs, promoting activation and upregulation of NLRP3 inflammasome and enhancing IL-1 β production in macrophages 205. Inhibition of PHB1 reduces IL-1 β expression and impairs clearance of *N. caninum*, highlighting the importance of PHBs in host defense. Further mechanistic studies investigating PHBs-NcGRA7 interaction at the IMM may provide insight into the regulation of NLRP3 upregulation and identify potential therapeutic targets for neosporosis.

Sepsis is a major global health burden, accounting for nearly 20% of worldwide deaths 206. Increasing evidence suggests that sepsis is associated with mitophagy dysfunction, a process that maintains mitochondrial homeostasis by removing damaged mitochondria generated by excessive inflammatory reactions 207. Mitophagy can suppress activation of the NLRP3 inflammasome, thereby limiting inflammatory damage 208. Clinical observations indicate that patients with higher levels of mitophagy exhibit improved prognosis, whereas impaired mitophagy contributes to inflammasome hyperactivation and worsened disease outcomes 207. PHB1, a core gene of the mitophagy network, is negatively correlated with sepsis severity and NLRP3 inflammasome expression. PHB1 deficiency increases cytosolic mitochondrial DNA levels, enhancing NLRP3 activation. Conversely, PHB1-mediated mitophagy suppresses NLRP3 inflammasome activation 207. These findings position PHB1 as a novel inflammasome regulator via mitophagy. In addition, PHB1 is abundant in the blood and behaves like an acute-phase reactant protein during sepsis 209. Overexpression of PHB1 preserves mitochondrial oxidative phosphorylation and upregulates the antioxidant capacity in cardiomyocytes undergoing inflammatory stress 209. Circulating PHB1 levels may serve as a potential biomarker of cardiac or organ dysfunction in patients with sepsis, offering a blueprint for the development of novel strategies to treat this condition.

### PHBs in clinical trials

Targeting PHB1 beyond pre-clinical models was evidenced by a first-in-man Phase I interventional clinical trial using Prohibitin Targeting Peptide 1 (Prohibitin-TP01) in patients with metastatic prostate cancer and obesity (ClinicalTrials.gov ID NCT01262664). Although this trial using Prohibitin-TP01, which is a 25-mer peptide that specifically binds to PHB1 in the white adipose vasculature, was terminated at the request of the principal investigator, this trial provides an example of growing interest in targeting PHBs for various disorders and signifies the potential of blocking peptides in addition to neutralizing antibodies and pharmacological agents to target PHBs *in vivo*. Future studies aimed at identifying specific amino acid post-translational modifications of PHBs will provide further opportunity to alter PHBs at specific subcellular locations that may lead to more targeted anti-infectious response depending on the microbe/PHB interaction. Additionally, based on the roles of PHBs during viral and bacterial infection, the discovery of new molecules involved in microbe/PHB interactions and PHB signaling upon infection provides additional drug targets to inhibit infectious diseases.

## CONCLUSIONS

PHB1 and PHB2 are multifunctional proteins that exhibit diverse functions within the cell, depending on their subcellular localization, including the mitochondria, plasma membrane, and nucleus. At the cell membrane, PHBs can serve as receptor proteins for viral and bacterial infection, promoting pathogenicity. In the mitochondria, PHBs are essential for mitochondrial integrity, dynamics and function. Notably, some viruses and bacteria have evolved strategies to exploit mitochondrial PHBs, mainly to support their replication or modulate host cell survival. Given these roles in viral and bacterial infection, PHB1 and PHB2 represent promising novel targets for the development of anti-infectious agents. Taking advantage of PHBs functions could offer a dual approach: inhibiting pathogenicity by restricting pathogen entry at the cell surface and disrupting pathogen-mediated manipulation of mitochondrial processes. These approaches could limit viral and bacterial pathogenicity and associated diseases.

## CONFLICT OF INTEREST

The authors declare no conflicts of interest.

## ABBREVIATIONS

CHIKV – Chikungunya virus

DENV – Dengue virus

EV-A71 – Enterovirus-A71

H1N1 – Influenza A virus subtype H1N1

HCV – Hepatitis C virus

HSV-1 – Herpes simplex virus 1

IFN – interferon

IMM – inner mitochondrial membrane

ISGs – interferon-stimulated genes

LCMV – Lymphocytic Choriomeningitis virus

mRNA – messenger RNA

mtDNA – mitochondrial DNA

Mtb – Mycobacterium tuberculosis

PHB – prohibitin

Opa1 – optic atrophy 1

OXPHOS – oxidative phosphorylation

Roc-A – Rocaglamide-A

ROS – reactive oxygen species

TCA – tricarboxylic acid cycle

TMEV – Theiler's mouse encephalomyelitis virus
